# Assessing Suicide Risk and Emotional Distress in Chinese Social Media: A Text Mining and Machine Learning Study

**DOI:** 10.2196/jmir.7276

**Published:** 2017-07-10

**Authors:** Qijin Cheng, Tim MH Li, Chi-Leung Kwok, Tingshao Zhu, Paul SF Yip

**Affiliations:** ^1^ HKJC Center for Suicide Research and Prevention The University of Hong Kong Hong Kong China (Hong Kong); ^2^ Department of Paediatrics & Adolescent Medicine LKS Faculty of Medicine The University of Hong Kong Hong Kong China (Hong Kong); ^3^ Institute of Psychology & Insititute of Computing Technology Chinese Academy of Sciences Beijing China

**Keywords:** suicide, psychological stress, social media, Chinese, natural language, machine learning

## Abstract

**Background:**

Early identification and intervention are imperative for suicide prevention. However, at-risk people often neither seek help nor take professional assessment. A tool to automatically assess their risk levels in natural settings can increase the opportunity for early intervention.

**Objective:**

The aim of this study was to explore whether computerized language analysis methods can be utilized to assess one’s suicide risk and emotional distress in Chinese social media.

**Methods:**

A Web-based survey of Chinese social media (ie, Weibo) users was conducted to measure their suicide risk factors including suicide probability, Weibo suicide communication (WSC), depression, anxiety, and stress levels. Participants’ Weibo posts published in the public domain were also downloaded with their consent. The Weibo posts were parsed and fitted into Simplified Chinese-Linguistic Inquiry and Word Count (SC-LIWC) categories. The associations between SC-LIWC features and the 5 suicide risk factors were examined by logistic regression. Furthermore, the support vector machine (SVM) model was applied based on the language features to automatically classify whether a Weibo user exhibited any of the 5 risk factors.

**Results:**

A total of 974 Weibo users participated in the survey. Those with high suicide probability were marked by a higher usage of pronoun (odds ratio, OR=1.18, *P*=.001), prepend words (OR=1.49, *P*=.02), multifunction words (OR=1.12, *P*=.04), a lower usage of verb (OR=0.78, *P*<.001), and a greater total word count (OR=1.007, *P*=.008). Second-person plural was positively associated with severe depression (OR=8.36, *P*=.01) and stress (OR=11, *P*=.005), whereas work-related words were negatively associated with WSC (OR=0.71, *P*=.008), severe depression (OR=0.56, *P*=.005), and anxiety (OR=0.77, *P*=.02). Inconsistently, third-person plural was found to be negatively associated with WSC (OR=0.02, *P*=.047) but positively with severe stress (OR=41.3, *P*=.04). Achievement-related words were positively associated with depression (OR=1.68, *P*=.003), whereas health- (OR=2.36, *P*=.004) and death-related (OR=2.60, *P*=.01) words positively associated with stress. The machine classifiers did not achieve satisfying performance in the full sample set but could classify high suicide probability (area under the curve, AUC=0.61, *P*=.04) and severe anxiety (AUC=0.75, *P*<.001) among those who have exhibited WSC.

**Conclusions:**

SC-LIWC is useful to examine language markers of suicide risk and emotional distress in Chinese social media and can identify characteristics different from previous findings in the English literature. Some findings are leading to new hypotheses for future verification. Machine classifiers based on SC-LIWC features are promising but still require further optimization for application in real life.

## Introduction

### Background

Suicide is the second leading cause of death in 15-29-year-olds globally and the first for this age group in China [1,2]. In addition to suicide as the most extreme action, more young people are suffering from emotional distress, which not only reduces their quality of life but also becomes a risk factor for severe mental disorder and suicide [3,4]. Therefore, early identification and intervention in emotional distress and suicidal thoughts are imperative for preventing suicide deaths.

To assess suicide risk and emotional distress, many tools have been developed and validated. Some examples of such tools include Adult Suicide Ideation Questionnaire [5], Suicide Probability Scale (SPS) [6], Depression Anxiety Stress Scales-21 (DASS-21) [7,8], and the recently developed Suicidal Affect-Behavior-Cognition Scale [9]. These tools often require respondents to either fill in a questionnaire or participate in a professional interview. However, distressed or suicidal people often have low motivation to seek help from professionals [10-12]. In addition, a recent study found that taking a suicide assessment may lead to negative affect changes on individuals with depressive symptoms [13]. From the suicide prevention point of view, a tool that can assess one’s suicide risk and emotional distress in a natural setting without costing his or her efforts and attention is preferable and can increase the opportunities for early identification and intervention.

### Previous Work

The wide use of Web-based social media has provided a natural setting where interpersonal communications can be well documented for studying suicide and mental health issues [14]. Cases of social media being used by individuals to express suicidal thoughts, look for suicide methods, or even live broadcast suicidal behaviors have been reported and studied in different countries [15-17] including China [18,19]. With Twitter and Facebook blocked in China, Sina Weibo (referred to as Weibo hereafter; Sina is a company name and Weibo literally means Microblog) is one of the most popular social media platforms among the Chinese population. According to China Internet Watch, Weibo had more than 313 million of monthly active users by the end of 2016 [20], which is close to the number of worldwide monthly active users of Twitter [21]. A recent study empirically demonstrated that Weibo users who have suicidal ideation or distressed mental states are very likely to tell others about their suicidal thoughts on Weibo [22]. This is in line with psycho-linguistic studies that see words or language as a meaningful marker to convey or predict different aspects of our minds [23].

Previous studies have demonstrated the potential to use social media data to assess suicide risk or depression in English [16,24-26]. There are relatively few studies on the same topic in Chinese, and only a handful of studies have explored the topic using Weibo data. These studies had several major limitations. First, some studies validated their machine learning models against human annotated suicide risk level [27,28]. The human annotators were often graduate students who were not systematically trained in suicide prevention. The validity of their annotation requires empirical examination [29]. Empirically validated assessment tools are a more rigorous way to validate machine classifier’s performance [24].

Second, most of the previous studies have artificially boosted the percentage of suicidal or depression cases in their total sample [30,31] or their classifiers were trained to distinguish extremely high suicidal cases from extremely low suicidal ones but excluding those in the middle [32]. Such study designs have difficulty being applied to real life scenarios, where people with different levels of risk are mixed, and suicidal people often count for a small proportion of the total population.

Last but not the least, previous Chinese studies have utilized a locally developed dictionary, namely, simplified Chinese micro-blog word count dictionary [33], for analyzing Weibo posts [32,34]. The advantage of the locally developed dictionary is that it might have a higher coverage of Chinese Web-based language. However, the disadvantage is that the results can hardly be compared with other countries’ studies that often use the standardized linguistic inquiry and word count (LIWC) dictionary [35]. More importantly, when previous work used the local dictionary to classify a Weibo user’s suicide risk, the classifiers’ performance showed a large space for improvement [32] or remained unclear [31]. In this case, it is worthy of empirical examination to find out whether using standardized LIWC dictionary can achieve comparable or even better performance than using a locally developed dictionary.

### Aim of the Study

This study aimed to explore whether computerized language analysis methods can be utilized to assess Chinese individuals’ suicide risk and emotional distress based on their Weibo posts. Specifically, we not only analyzed what Simplified Chinese-Linguistic Inquiry and Word Count (SC-LIWC) categories were associated with suicide risk or emotional distress but also applied machine learning method to automatically classify whether a social media user was having suicide risk or emotional distress. We examined the computerized markers’ performance against conventional self-assessment tools to evaluate their utility.

## Methods

### Data Collection

A Web-based survey of Weibo users was conducted to assess the respondents’ suicide risk and emotional distress (ie, depression, anxiety, and stress). The invitation letter to participate in this survey was widely sent out to general Weibo users by various promotion activities. For a Weibo user to be eligible for the study, she or he had to be 18 years or older (by self-report). A 30 Renminbi incentive for each complete survey was provided to boost the respond rate. With the respondents’ consent, their Weibo posts that were posted in the public domain during the 12 months before the survey were downloaded by calling Weibo API. The survey fulfilled the *Checklist* for Reporting Results of Internet E-Surveys (CHERRIES) checklist and details of the procedure have been reported in previous publications [22,32]. In addition, when multiple survey feedback were submitted from the same Internet protocol addresses, only the first submission was used to avoid duplicate participation. In contrast to a previous study [32], this study excluded those who posted nothing throughout the 12 months but not those who posted fewer than 100 posts. Eventually, data provided by 974 respondents remained for further analyses.

The study has obtained ethical approvals from the Human Research Ethical Review Committee at the University of Hong Kong and the Institute Review Board of the Institute of Psychology at the Chinese Academy of Sciences.

The survey measured respondents’ suicide probability score, depression, anxiety, stress, and Weibo suicide communication (WSC) as the outcome variables. In addition, the respondents’ Weibo posts language features were extracted as independent variables or features for machine learning. The details of how those data were obtained are elaborated in the following subsections.

#### Suicide Probability

The Chinese version of the SPS was adopted to assess the respondents’ suicide probability. The SPS was originally developed in the United States and then translated and validated in China [36,37]. The Cronbach alpha coefficient of the scale in our study was .749.

#### Depression, Anxiety, and Stress

The Chinese version of the DASS-21 was used to measure the respondents’ emotional distress, which has been validated in China and has shown good construct validity and criterion-related validity [7,8,38]. The scale includes 3 subscales to measure depression, anxiety, and stress, respectively. In our study, the Cronbach alpha coefficient was .859 for the depression subscale, .767 for the anxiety subscale, and .821 for the stress subscale.

#### Weibo Suicide Communication (WSC)

WSC was measured by a single-item question on whether or not the respondent had told others via Weibo in the past 12 months that he or she wanted to kill himself or herself. Given Weibo’s multiple functions, WSC can be delivered by publishing Weibo posts, sending private messages to others, or expressing suicidal thoughts in a group chat. For this question, the respondent was not limited to any particular type of Weibo communication.

#### Language Features

Weibo posts were segmented using the Stanford word segmenter [39] that resulted in 349,374 words and phrases. Thereafter, the SC-LIWC [33] dictionary was applied to count the appearance of each category of words in every respondents’ Weibo posts. The SC-LIWC dictionary includes 7450 words that are grouped into 71 categories, including 7 main linguistic or psychological categories and 64 subcategories. In addition, the total number of words or phrases that each respondent published in the 12 months was counted as the 72nd category. Scores of the SC-LIWC categories were counted as percentages of the total number of words.

### Data Analysis

#### Simplified Chinese-Linguistic Inquiry and Word Count (SC-LIWC) Categories as Markers

Five rounds of logistic regression analysis were applied by including the 5 suicide risk factors (SPS, depression, anxiety, stress, and WSC) as dependent variables, respectively. Binary classifications of the 5 risk factors were used in the logistic regression analyses. We followed previous studies to use the total score of 80 as the cut-off for the SPS [6,36,40], 10 for severe depression, 7 for severe anxiety, and 12 for severe stress [7,8,38] to categorize the respondents to “at-risk” and “others” groups, respectively. As for WSC, the “at-risk” group is defined as exhibiting WSC, whereas the “others” group as not exhibiting WSC in the past 12 months. For each suicide risk factor, all 72 linguistic features of SC-LIWC were entered as independent variables to a stepwise regression for feature selection at a significance level of .05.

#### Automatic Machine Classifiers as Markers

The support vector machine (SVM), a supervised machine learning model, was employed to build algorithms for automatically classifying whether a Weibo user is having suicide risk or emotional distress. SVM is a well-known and highly effective approach yielding high accuracy in affect and sentiment analysis in computer science [41]. The scores of the SC-LIWC categories were included as the features for SVM classification.

SVM classification also requested the outcome variable to be binary, which was consistent with the logistic regression analysis. R version 3.0.0 (The R Project for Statistical Computing) with package “e1071” was used to conduct SVM training [42].

Furthermore, since our previous examination found that exhibiting WSC can be explained by suicidal ideation and negative affectivity [22], we further used the WSC variable as a filter. Specifically, we only included those respondents who reported having WSC in the survey and then ran the SVM training solely on those respondents. It was expected that this screening method could further improve the performance of the SVM model. All the classification results were generated with leave-one-out cross validation that was found to be able to provide an almost unbiased estimator of the generalization properties of statistical models [43,44].

Receiver operating characteristic (ROC) curve analysis was operated for analyzing and comparing the diagnostic accuracy of the SVM classifications for the 5 risk factors. The primary outcomes of the study were the area under the ROC curves, sensitivities, and specificities of the SVM classifiers.

**Table 1:** Logistic regression on total respondents (N=976).

Dependent variable	SC-LIWC^a^ category	Examples in Chinese with English translation^b^	Estimate	Standard error	Odds ratio	*P* value
**Suicide probability scale**						
	Personal pronoun	你 (you [as singular])、她们 (they [as females])	0.17	0.05	1.18	.001
	Verb	分享 (share)、开车 (drive)、听 (listen)	−0.24	0.06	0.78	<.001
	Prepend^b^	之中 (among)、以上 (above)、为止 (until)	0.40	0.16	1.49	.02
	Multifunction^c^	的 (of or target or possessive or adjectival suffix)、有 (have or own or possess or exist)、是 (yes or indeed or right or to be or demonstrative pronoun or this or that)	0.12	0.06	1.12	.04
	Total length (every 1000 words)		0.007	0.003	1.007	.008
**Weibo suicide communication**						
	Personal pronoun	他 (he)、大家 (all)、你们 (you [as plural])	0.14	0.05	1.15	.004
	Third-person plural	她们 (they [as females])、他们 (they [as males])	−3.88	1.95	0.02	.047
	Work	工厂 (factory)、面试 (interview)、薪水 (salary)	−0.34	0.13	0.71	.008
**Depression**						
	Second-person plural^b^	你们 (you [as plural])、汝等 (you [as plural])	2.12	0.82	8.36	.01
	Work-related	工厂 (factory)、面试 (interview)、薪水 (salary)	−0.58	0.20	0.56	.005
	Achieve-related	擅长 (good at)、尽责 (responsible)、高手 (master)	0.52	0.18	1.68	.003
**Anxiety**						
	Work-related	工厂 (factory)、面试 (interview)、薪水 (salary)	−0.26	0.11	0.77	.02
**Stress**						
	Third-person plural	她们 (they [as females])、他们 (they [as males])	3.72	1.81	41.33	.04
	Second-person plural^b^	你们 (you [as plural])、汝等 (you [as plural])	2.40	0.85	11.00	.005
	Health-related	失眠 (insomnia)、医生 (doctor)、运动 (exercise)	0.86	0.30	2.36	.004
	Death-related	亡故 (die)、自杀(suicide)、遗嘱 (will)	0.96	0.38	2.60	.01

^a^SC-LIWC: Simplified Chinese-Linguistic Inquiry and Word Count.

^b^The translations were adopted from ZDIC [45].

^c^The category only applies to Chinese but not English.

## Results

### SC-LIWC Categories as Markers

Table 1 presents the SC-LIWC categories that showed independent effects on differentiating those at-risk ones from the other respondents in the final regression model after stepwise selection. *P*<.05 was adopted as the cut-off for statistical significance. For example, as shown in Table 1, a 1% increase in usage of any pronoun would increase the risk of having high level of SPS by 18% (odds ratio, OR=1.18, *P*=.001). By contrast, more frequent use of verb was associated with lower risk (OR=0.78, *P*<.001). In short, Weibo users with high suicide probability were marked by a higher usage of pronoun, prepend and multifunction words, a lower usage of verb, and a greater total word count. The markers of the other 4 risk factors showed more commonalities. For example, second-person plural was positively associated with severe depression and stress, whereas work-related words were negatively associated with WSC, severe depression, and anxiety. Meanwhile, some special characteristics were associated with the different risk factors. Third-person plural was found to be negatively associated with WSC but positively with severe stress. Achievement-related words were positively associated with depression, whereas health- and death-related words were positively associated with stress.

### Automatic Machine Classifiers as Markers

**Figure 1:**
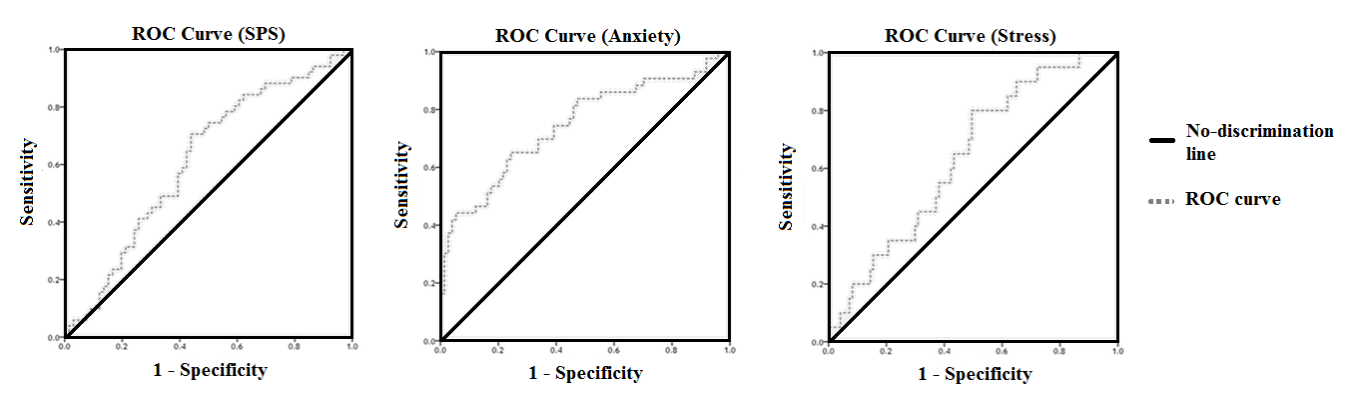
Receiver operating characteristic (ROC) curves of the supportive vector machine (SVM) classification for high suicide risk, severe anxiety, and stress among participants who had Weibo suicide communication.

Table 2 demonstrates the AUCs, sensitivities, and specificities of the SVM classifiers for whether a Weibo user was at one of the five types of risk. There were no significant AUCs for the SVM classifiers of the total respondents for the 5 risk factors. However, when we filtered out those non-WSC respondents, SVM classification significantly identified those with high suicide probability or severe anxiety. The classification for severe stress was marginally significant, whereas the one for severe depression was still not significant. The performance characteristics of the 3 significant and marginally significant SVM classifiers are shown in Figure 1 as summarized by ROC curves.

**Table 2:** Receiver operating characteristic (ROC) curve analyses on supportive vector machine (SVM) classifiers of Weibo users’ suicide probability and emotional distress.

Outcome Variables	n (%)	AUC^a^ (95% CI)	*P* value	Sensitivity^b^ (%)	Specificity (%)
For all respondents (N=976)					
Had Weibo suicide communication	117 (12)	0.56 (0.50-0.61)	.06	61	49
High suicide risk (SPS≥80)	190 (19)	0.48 (0.44-0.53)	.43	64	32
Severe depression (DASS^c^-Depression score>10)	49 (5)	0.47 (0.38-0.55)	.41	63	33
Severe anxiety (DASS-Anxiety score>7)	140 (14)	0.45 (0.40-0.50)	.06	58	32
Severe stress (DASS - Stress score > 12)	45 (5)	0.47 (0.39-0.56)	.52	64	33
For respondents who had Weibo suicide communication (N=117)					
High suicide risk (SPS^d^≥80)	51 (44)	0.61 (0.51-0.72^)^	.04	65	58
Severe depression (DASS-Depression score>10)	23 (20)	0.57 (0.42-0.72)	.31	65	50
Severe anxiety (DASS-Anxiety score>7)	43 (37)	0.75 (0.65-0.84)	<.001	70	66
Severe stress (DASS-Stress score>12)	20 (17)	0.64 (0.52-0.76)	.05	65	57

^a^AUC: area under the curve.

^b^Sensitivity and specificity at the optimal cut-off were reported.

^c^DASS: Depression Anxiety Stress Scales.

^d^SPS: Suicide Probability Scale.

## Discussion

### Principal Findings

The study demonstrates the utility of natural language processing (NLP) methods to assess suicide risk and emotional distress in Chinese social media. Significant associations between certain SC-LIWC categories and suicide risk or emotional distress were identified. In addition, automatic machine classifiers achieved satisfying accuracy when classifying suicide probability and anxiety level among those who had expressed suicidal thoughts to others via Weibo. However, the classifiers’ performance on classifying depression and stress levels needs to be improved at large. The study sheds light on the potentials and challenges of developing automatic computerized program to assess mental risk based on natural language processing in Chinese. Although the study design is data-driven rather than hypothesis-driven, we will further discuss some key results by relating them to existing theories and previous research findings.

### SC-LIWC Categories as Markers

It is noteworthy that this study did not find a significant association between first person singular pronouns (ie, I, me, and my) and suicide risk or emotional distress, which is inconsistent with a number of previous studies [46-48]. The phenomenon might be related to the fact that first person singular in Chinese conveys an ambiguous meaning, which not merely refers to the addresser as himself or herself but also shows a tendency toward putting him or her in a whole community that the addresser belongs to, thus bringing the addresser a sense of empathy and friendly interpersonal relationship [49]. In this case, the use of first person singular in Chinese not necessarily indicates a self-focus mind and may not be able to mark suicide risk or emotional distress like in English. In addition, it is of note that a recent study examining linguistic characteristics of suicide related Tweets found that the first person pronouns can differentiate strongly-concerned Tweets from safe-to-ignore Tweets [48]. However, they have excluded possibly concerning Tweets from their original dataset that made their results not directly comparable with ours.

In addition, those Chinese social media users with greater levels of depression and anxiety were more likely to write more of second person plural pronouns in their public posts. This suggests that they preferred referring or talking to a group of others directly in their posts, which was potentially inviting a direct communication with others. Suicide prevention professionals may make best of this opportunity to proactively engage with at-risk ones and offer help and support. The findings on third person plural’s association with the outcome variables were not consistent. While being negatively associated with WSC, it showed positive association with stress. No previous literature reported similar findings. Nevertheless, the inconsistency suggests that those having severe stress might be different from those having WSC in terms of how they relate themselves with third parties.

Death-related words were associated with severe stress but not suicide probability. This finding is different from previous findings in English that suicidal poems talked about death-related more often [47], as well as a Japanese study that showed tweeting “want to commit suicide” could predict suicidal ideation and attempt [16]. The divergence might be related with the different study design: our study compares people with greater suicide risk to those with lower risk, whereas the previous studies did comparisons either between those suicides deceased and alive nonsuicidal ones, or between those with history of suicide attempts with those without. Furthermore, our findings suggest that the Chinese Weibo users at high suicide probability might express their suicidal thoughts implicitly, rather than using words of death and suicide, in the public domain. By contrast, those with severe stress but not necessarily planning to kill themselves were more likely to disclose their emotional distress by using words relating to death and suicide.

The usage of achievement-related words was positively associated with depression. This is in line with previous studies that found achievement-oriented to be often confounded with depressive symptoms [50-53]. However, a previous machine learning study based on Twitter users in the United States found that the greater usage of achievement-related words in Tweets was associated with being nonsuicidal [24]. Although the US study did not examine depression, the differences between our findings with theirs warrant more studies on the cross-cultural differences regarding the relationship between achievement and suicide or emotional distress.

The use of work-related words was negatively associated with depression, anxiety, and WSC. The phenomenon might be interpreted from two different angles. First, it suggests that those distressed individuals were likely unemployed, which is known to be a risk factor for suicide and emotional distress. The alternative interpretation is that those who were more motivated by their work would demonstrate more positive mental states.

### Automatic Machine Classifiers

The results of the machine learning analysis demonstrated the challenges of automatically assessing one’s suicide probability or emotional distress by NLP. This is related to the fact that prevalence of the outcome variables among the general population is somewhat low. However, by adding a filter of WSC, our machine classifiers’ performance has been improved, especially that of suicide probability and anxiety. This is because WSC was found to be highly correlated with the outcome variables [22], which helps to boost the prevalence of the outcome variables among the filtered population. As discussed in the Introduction section, previous studies often artificially boosted the percentage of suicidal or depression cases in their total sample [30,31] or purposely excluded those with medium level of risk from the sample [32]. Different from those studies, the filter of WSC used in this study indicated real behaviors of expressing one’s suicidal thoughts via Weibo to others. In real life scenarios, it is feasible to encourage those who have read or received Weibo posts or messages about suicidal thoughts to refer those posts to our algorithms for further assessment.

There is certainly room to further optimize the machine classifiers’ sensitivity and specificity. Braithwaite and colleagues’ recent study using Twitter data in the United States adopted a similar study design as the presented study but their classifiers outperformed ours in terms of accuracy [24]. Braithwaite and colleagues used different scales to measure suicide risk and different machine learning model to develop their classifiers. It is worthy of our future efforts to find out whether following their approaches can improve the classification performance in the Chinese settings as well. Nonetheless, the performance of the suicide probability classifier and anxiety classifier with filter is promising. It is important that applying the classifiers to review and assessing the posts is much more efficient, convenient, and less costly compared with doing it manually or inviting those Weibo users to conduct questionnaire survey.

### Limitations

A few limitations of the study should be noted. The machine classifiers developed by this study need to be further optimized, especially the classifiers of depression and stress. More replicative studies are still needed to examine the transferable validity of our research findings.

The Web-based survey adopted a random sampling approach. However, the respondents may have been self-selected because of their interest in psychological research. Nonetheless, we have compared the basic demographic characteristics (ie, age and gender) of the survey respondents with the general Weibo users and found no significant differences [32].

Last but not the least, the study was conducted in a data-driven manner that led to the results being less structured and some results difficult to interpret. In fact, the study has brought up more questions and new hypotheses for future studies rather than verifying or confirming existing theories.

### Implications and Future Research

To apply the language markers and automatic classifiers in real life, we would suggest Weibo users to be more cautious when reading a post or message about suicide. When suspecting someone might be at risk, they can refer the person’s Weibo account to our classifiers that will automatically screen that person’s public posts and further assess his or her conditions. It will be beneficial if a longitudinal study can be carried out to apply the algorithms developed by this study to screen and assess Weibo posts continuously and provide the results to suicide prevention professionals for double check and follow-up. In turn, the experts’ feedback and follow-up results should be fed back to the model’s developers for optimization [54].

Some social media platforms, such as Facebook and Instagram, have developed “report” functions to allow users to flag those that are expressing suicidal thoughts. The report will be manually reviewed by in-house reviewers to decide whether the flagged person is indeed at risk. If automatic classifiers such as the ones developed by this study can be integrated into such kind of Web-based report function, it will improve review efficiency and better empower social media platforms and users to contribute to suicide prevention. As social media are rapidly penetrating into our daily life, the opportunities for detecting and engaging distressed individuals via social media should not be missed.

### Conclusions

This study demonstrates that natural language in social media can be utilized as markers to differentiate those at-risk individuals from the general population and that the language markers are culturally sensitive. The automatic computer program shows potential for aiding human watchers to assess suicide probability and anxiety by improving the assessment efficiency but not compromising significant accuracy.

## Abbreviations

AUC: area under the curve
CHERRIES: Checklist for Reporting Results of Internet E-Surveys
DASS-21: Depression Anxiety Stress Scales-21
LIWC: Linguistic Inquiry and Word Count
ROC: receiver operating characteristic
SC-LIWC: Simplified Chinese Linguistic Inquiry and Word Count
SPS: Suicide Probability Scale
SVM: supportive vector machine
WSC: Weibo suicide communication
